# Reflex phase of regulation of pancreatic secretion in poultry after feed intake is associated with gustatory sensations, and neurohumoral—with nutritional value

**DOI:** 10.3389/fphys.2024.1341132

**Published:** 2024-03-12

**Authors:** Vladimir G. Vertiprakhov, Vladimir I. Trukhachev, Nadezhda A. Sergeenkova

**Affiliations:** Timiryazev Russian State Agrarian University—Moscow Agrarian Academy, Moscow, Russia

**Keywords:** pancreatic juice, broiler chickens, postprandial secretion, digestive enzymes, feed flavor

## Introduction

Poultry farming is the fastest growing sector of agriculture. Balanced diets are an important factor for realisation of the genetic potential of birds. The pancreas plays an important role in the digestive process (Vertiprakhov, Grozina, Fisinin, 2023). To improve nutrition, it is necessary to clearly represent the physiological needs of the body of broiler chickens, based on the bird’s preference for the taste and nutritional properties of the feed. The question of the ability of the pancreas to adapt to the nature of nutrition in animals has been debated to date. There is a large amount of experimental data indicating the ability of the pancreas to change the composition of its secretion accordingly to the nature of the food consumed, including in poultry (Batoev, 2018; Fisinin, Vertiprakhov, Kharitonov, Grozina, 2019). The possibility of urgent change of enzymatic spectrum of secretion depending on the type of taken food is postulated. This problem is of particular relevance due to the intensive development of poultry breeding and the need to use the genetic potential of modern lines and crosses to the fullest. It is impossible to do it without fundamental knowledge about functional activity of digestive system. Studies of regulation of secretory function of the pancreas should be conducted under conditions close to normal physiological conditions. Attempts to obtain pure pancreatic juice were aimed at implanting a cannula into the pancreatic duct (Degolier et al., 1999), but this method has not found wide application. Studies of digestive enzymes in the intestine of poultry (Ren et al., 2012; Sun et al., 2013) do not claim to decipher the mechanisms of regulation of pancreatic secretory function. In the present work we express an opinion based on the results of experiments on broiler chickens with chronic fistula of the pancreatic duct, whereby pancreatic juice was obtained during the period of experiments and directed to the intestine during the rest of the time.

## Secretory function of the pancreas of broiler chickens during the day

**FIGURE 1:**
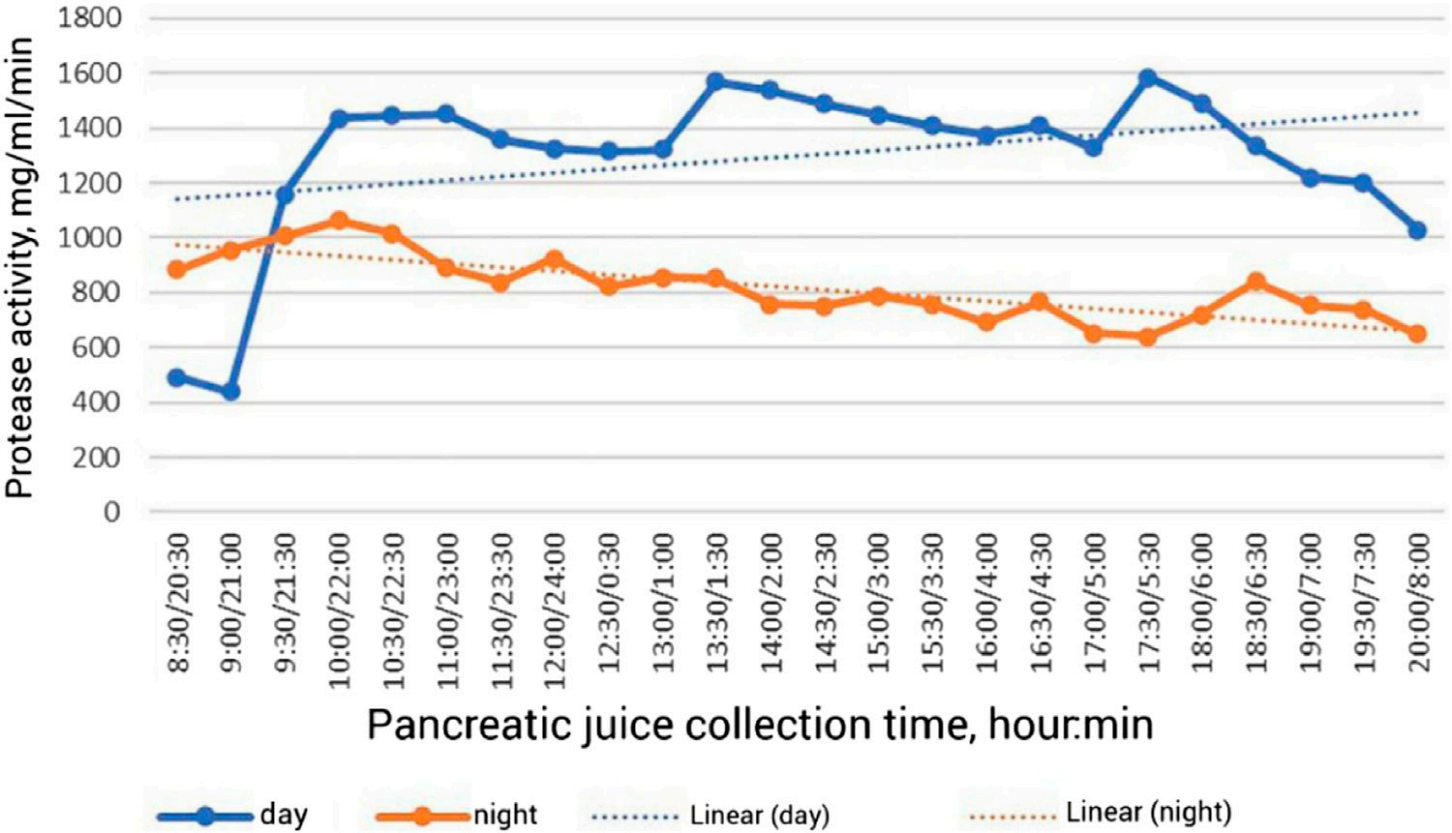
Dynamics of proteolytic activity of pancreatic juice in broiler chickens during the day (Vertiprakhov, 2022).

There are few data on the secretory function of the pancreas in birds in the scientific literature. This is explained by a certain methodological difficulty in obtaining pancreatic juice in chronic experiment. A unique surgical operation on implantation of the pancreatic duct into an isolated section of the intestine was developed by Batoev (1970); he was the first to show changes in the secretory function of the pancreas in geese, ducks and chickens. It was found that for a day per 1 kg of weight of chickens and ducks secretes an average of 28 mL, in geese–16 mL of pancreatic juice (Batoev, 2001). In broiler chickens, 31.4 mL of pancreatic juice is secreted per 1 kg of live weight per day. Pancreatic juice is secreted continuously during the day in birds. A strong stimulant of pancreatic juice secretion is the intake of feed and water. In broiler chickens in the morning hours in the postprandial phase, the amount of juice increases 2 times compared to the initial value (Figure 1) (Vertiprakhov and Egorov, 2016; Vertiprakhov, 2022). In the daytime in the postprandial period, the increase is 13.3% compared to the preprandial period. In the evening period, the increase in juice secretion with the baseline amounted to 14.3%, but there was a rapid decline in secretion. In the night period without feed intake in contrast to the daytime, a 2-fold decrease in pancreatic juice secretion was observed. The curve of pancreatic juice secretion during the night period shows a cyclicity of 180–240 min. The cyclic increase in secretion is associated with motilin, which has been found in the blood of dogs (Lee et al., 1986).

## Changes in the activity of pancreatic enzymes during the day

Studies on broiler chickens with chronic pancreatic duct fistula were fully consistent with the digestive process under physiologic conditions.

The results showed that during the first hour of the postprandial phase in the morning hours the activity of proteases increases 3.3 times (Figure 1), amylase–3.2 times, lipase–2.1 times (Vertiprakhov, 2022). A significant level of enzyme activity is maintained until the next feeding, after which the activity of proteases increases by 19.0%, amylase—by 17.0%, lipase—by 42.0% compared to the initial level before feeding. After the evening meal, protease activity increases 19.2%, amylase activity increases 21.1%, and lipase activity increases 17.4% over baseline levels. All pancreatic enzymes show an increase in activity during the day period, which is due to feed and water intake. The opposite trend is observed during the night period, resulting in a 37.0% decrease in protease activity, a 25.0% decrease in amylase activity and a 23.0% decrease in lipase activity compared to the average daily level. Consequently, the activity of pancreatic enzymes determines the adaptation of digestion to the feed consumed, therefore, the study of enzymatic activity is of fundamental importance in the study of the secretory function of pancreas, and the average level of basal secretion, which is maintained at night in the interprandial period is a criterion of long-term adaptation of digestion to the composition of the diet. Along with the activity of enzymes in the postprandial phase, the content of total protein, fructosamine, phosphorus, zinc, and copper in pancreatic juice changes (Grozina, 2019). It was found that the amount of proteolytic enzymes in the pancreatic juice of chickens increased after feed intake, as well as the amount of total protein, while the activity of pancreatic amylase increased sharply (by 10.8%) immediately after feed intake, followed by minor spikes within 3 h after feeding. Lipase activity increased sharply (by 52.8%) immediately after feeding, then it also changed in a jump-like manner. The content of fructosamine in pancreatic juice also increased slightly. Thus, stimulation of pancreatic secretion is realized during the period of feed and water intake.

## Regulation of pancreatic secretion in broiler chickens

The pancreas secretes secretion continuously. In both the interdigestive and postprandial states, pancreatic secretion is regulated by nervous and hormonal action as well as by neurohormonal interactions (Pavlov, 1951). Batoev (2001) and Berdnikov (2009) made a great contribution to the study of the regulation of pancreatic secretory function in chickens, ducks and geese. It was confirmed that feeding serves as a powerful stimulus of pancreatic secretion. The period of the complex-reflex phase of pancreatic regulation in broiler chickens begins in the first 30 min and lasts 90 min from the moment of feeding. At this time regulation of the pancreas is provided by complex-reflex, and the feed consumed by the bird is in the goiter or stomach, and pancreatic juice is intensively separated under the influence of impulses coming by parasympathetic fibers from the digestive center in the medulla oblongata, where they come from the receptors of the oral cavity. In this case, depending on the strength of the stimulus acting on the receptors of the oral cavity, the level of pancreatic secretion changes, i.e., this period is conditioned by the gustatory qualities of the feed.

Studies on chickens and other birds have shown that the avian gustatory system consists of taste buds that are not assembled into papillae and are located mainly (60%) in the upper palate, hidden in the crevices of the salivary ducts. Chickens appear to have an acute sense of taste that allows them to distinguish food amino acids, fatty acids, sugars, quinine, Ca and salt among others. Recently, an avian chemosensory system linked to the enteroendocrine system has been discovered in the gastrointestinal tract and hypothalamus that mediates a dialog between the gut and the brain relevant to the control of food intake. (Niknafs, Roura, 2018). Celed-Shoval, Druyan, Uni (2015) experimental data using real-time PCR showed the expression of chicken taste receptor genes. Expression of these genes suggests the involvement of taste pathways for the perception of carbohydrates, amino acids and bitter compounds in the chicken GI tract. Umami taste is one of the five basic taste qualities, along with sweet, bitter, sour, and salty, and is determined by several l-amino acids and their salts, including monocalic l-glutamate (MPG) (Yoshida, Kawabata, Kawabata, Nishimura, Tabata, 2015). The secretory response of the pancreas to the dietary supplement “Garlic” with allicin under experimental conditions with chickens implanted with chronic pancreatic duct fistulas was that amylolytic and proteolytic activities increased in 1 mL in chickens during the experimental period (8 days). However, there is no significant change in total juice volume in 180 min (Vertiprakhov, Grozina, 2019). Thus, the presence of receptors in the bird’s oral cavity is the beginning of a reflex arc that ends in the medulla oblongata (digestive center) and determines the complex-reflex phase of digestive regulation, which lasts from 0 to 60–90 min after feeding in broiler chickens (Vertiprakhov, 2022). The neurohumoral phase of regulation of pancreatic secretion begins 90 min after feeding (Vertiprakhov and Borisenko, 2019), which is associated with secretin, which is released during the entry of acidic foods from the stomach into the intestine, stimulates pancreatic juice secretion. In birds such as duck, the direct mode needs additional help from a peptide that activates pituitary receptors (Wang and Cui, 2007). It is known (Chei, Chang, 2003) that secretin is a hormone regulating exocrine fluid and bicarbonate secretion by the pancreas, gastric acid secretion and gastric motility. Secretin release and action are regulated by hormonal-hormonal and neurohormonal interactions. The vagus nerve, especially its afferent pathway, plays an essential role in the physiologic action of secretin. The hormone cholecystokinin promotes the secretion of pancreatic enzymes. Cholecystokinin (CCK), initially involved in humoral secretion of pancreatic enzymes through direct action on CCK acinar receptors, is also essential for the mechanism of enteropancreatic reflexes. In the brainstem, vago-vagal enteropancreatic reflexes can be modulated by incoming signals from higher brain centers, particularly the hypothalamic-cholinergic system, in tonic stimulation of preganglionic neurons of the dorsal motor nucleus of the vagus nerve, which influences pancreatic function (Singer, Niebergall-Roth, 2009). The question of the role of pancreatic enzymes in animal blood in the regulation of pancreatic secretion is still debatable. The hypothesis of Laporte and Tremolieres (Laporte, Tremolieres, 1971) is interesting in this respect. Trypsin and chymotrypsin in the duodenum were found to have a negative effect on pancreatic enzyme secretion in rats. In addition, the feedback mechanism is neurally mediated, including the cholinergic pathway (Lui, May, Miller, Ouyang, 1986). Experiments conducted on chickens showed that 60 min after feeding, serum trypsin activity increased, while amylase and lipase activities remained at their respective preprandial levels. A correlation was found in the postprandial period: trypsin in the postprandial period increased simultaneously in intestine and blood, parallel to nitric oxide, indicating the participation of pancreatic enzymes in the regulation of pancreatic secretory function in poultry (Fisinin, Vertiprakhov, Titov, Grozina, 2018; Vertiprachov, Ovchinnikova, 2022). The strong positive correlation between trypsin activity in pancreas or pancreatic juice and chicken blood serum may be a starting point for further studies on the functions of circulating trypsin, which may include regulation of pancreatic exocrine activity and other vital functions (Vertiprakhov, Grozina, Fisinin, Egorov, 2018; 2021).

Pituitary adenylate cyclase-activating polypeptide (PACAP), a member of the secretin/glucagon/VIP family, has potent effects on pancreatic secretion in mammals (Vaudry et al., 2013). In experiments with purified peptide from rat pancreatic juice, the hypothesis that purified peptide is partially responsible for the humoral control of pancreatic enzyme secretion in response to food intake was confirmed (Fukuoka, Tsujikawa, Fushiki, Iwai, 1986). Thus, it can be considered that the most subtle adaptation of enzymes to the composition of the diet occurs in the neurohumoral phase of regulation of pancreatic secretion, when nutrients coming from the stomach into the duodenum cause the release of cholecystokinin, peptides, which enter the blood, reach the digestive center in the medulla oblongata and from there is an impulse to the pancreas, which secretes secretion with a certain enzyme activity, adequate to the composition of the diet.

## Conclusion

Thus, a powerful stimulus of pancreatic secretion is the ingestion of food and water, which during the complex-reflex phase of regulation is more affected by the increase in enzymatic activity depending on the basal level of secretion, which is adapted to the composition of the diet through taste receptors in the oral cavity, but this does not always have a clear direction. Therefore, the complex-reflex phase of regulation of pancreatic secretion depends on food flavor. The most perfect type of adaptation of the pancreas to the composition of the diet is the enzymatic adaptation during the period when partially digested food leaves the stomach and enters the duodenum. At this point, enzymes are clearly adapted to the nutritional value of the food. Therefore, studying the activity of digestive enzymes 60 and 120 min after ingestion is a key to assessing the flavor and nutritional properties of food and a prospect to offer chickens a more attractive feed to enhance metabolism and productivity (Perepelkina et al., 2012; Vertiprakhov et al., 2016; Liu et al., 2018).
